# Supplementary material for: The Contribution of Extruded and Fermented Wheat Bran to the Quality Parameters of Wheat Bread, Including the Profile of Volatile Compounds and Their Relationship with Emotions Induced for Consumers
Source: Foods. 2021 Oct 18;10(10):2501. doi: 10.3390/foods10102501 (PMC8535695; doi:10.3390/foods10102501)
Supplement: Supplementary file 1 [file foods-10-02501-s001.zip › Supplementary file 1. Characteristics of extruded and fermented wheat bran.pdf]

Table 1. Acidity, microbiological parameters, and sugar concentration in processed wheat bran.

Samples	pH		TTA, °N		Lactic Acid Content, g/100 g		LAB	M/Y	TBC	TEC	Fructose	Glucose	Sucrose	Maltose
	Duration of Fermentation, h				L(+)	D(−)	log10 CFU/g				g/100 g			
	0	24	0	24										
$W_{ex130/25/Lpl}$		4.22 ± 0.02c		3.90 ± 0.10b	0.423 ± 0.009c	0.322 ± 0.014a	8.46 ± 0.11d	4.29 ± 0.07b	8.63 ± 0.12ab	nd	nd	nd	nd	nd

W—wheat bran; Lu—fermented with *L. uvarum*; $_{ex130/screwspeed25}$—extruded at 130 °C and 25 rpm; TTA—total titratable acidity; LAB—lactic acid bacteria; M/Y—mould and yeast count; TBC—total bacteria count; TEC—total enterobacteria count; CFU—colony-forming units; nd—not detected; −—not analysed. Data expressed as mean values (n = 5) ± standard deviation (SD). a–e—means within a lines with different letters are significantly different ($p \leq 0.05$)

Table 2. Amino acid concentration (g/100 g) in processed wheat bran.

Samples	Asp	Glu	Asn	Ser	His	Gly	Thr	Arg	Ala	Tyr	Cys	Val	Met	Trp	Phe	Ile	Leu	Lys	Pro
$W_{ex130/25/Lu}$	0.48 ± 0.04a	1.47 ± 0.08a	nd	0.26 ± 0.02a	0.11 ± 0.01a	0.26 ± 0.02a	0.26 ± 0.02a	0.27 ± 0.02a	0.23 ± 0.02a	0.17 ± 0.01a	0.40 ± 0.03ab	0.34 ± 0.03a	0.13 ± 0.01a	0.29 ± 0.02a	0.22 ± 0.02a	0.32 ± 0.03a	0.11 ± 0.01a	0.34 ± 0.03b	0.24 ± 0.02b

W—wheat bran; Lu—fermented with *L. uvarum*; $_{ex130/screwspeed25}$—extruded at 130 °C and 25 rpm; nd—not detected; Asp—aspartic acid; Ala—alanine; Gly—glycine; Val—valine; Leu—leucine; Ile—isoleucine; Thr—threonine; Ser—serine; Pro—proline; Asn—asparagine; Met—methionine; Glu—glutamine; Phe—phenylalanine; Lys—lysine; His—histidine; Arg—arginine; Tyr—tyrosine; Trp—tryptophan; Cys—cysteine. Data expressed as mean values (n = 5) ± standard deviation (SD). a–f—means within a lines with different letters are significantly different ($p \leq 0.05$).

Table 3. Biogenic amines concentration (mg/kg) in processed wheat bran.

Samples	PUT	CAD	HIST	SPRM	TYR	PHE	SPRMD
$W_{ex130/25/Lu}$	91.3 ± 2.1a	33.8 ± 2.1a	9.2 ± 0.3a	35.9 ± 2.7b	nd	nd	nd

W—wheat bran; Lu—fermented with *L. uvarum*; $_{ex130/25}$—extruded at 130 °C and 25 rpm; PUT—putrescine; CAD—cadaverine; HIST—histamine; SPRM—spermine; PHE—phenylethylamine; TYR—tyramine; SPRMD—spermidine; nd—not detected. Data are represented as means (n = 5) ± SE. a–f—mean values within a lines denoted with different letters are significantly different ($p \leq 0.05$).

Table 4. Mycotoxin concentration (μg/kg) in processed wheat bran.

Samples	AOH	AME	17-DMAG	15-DON	DON	D3G	15ACS	ENN A	ENN A1	FB1	FB2	MEL	STC	OTB	OTA	T-2	HT-2	FUSX	Neo	AFB1
$W_{ex130/25/Lu}$	1.31 ± 0.12c	1.4 ± 0.09d	0.78 ± 0.06b	nd	19.9 ± 0.14a	0.44 ± 0.02b	1.81 ± 0.15b	1.29 ± 0.09a	0.26± 0.02a	0.08± 0.01a	nd	0.02± 0.01a	1.27± 0.09e	nd	nd	nd	1.17± 0.06d	nd	0.05± 0.02a	nd

W—wheat bran; Lu—fermented with *L. uvarum*; $_{ex130/screwspeed25}$—extruded at 130 °C and 25 rpm; AOH—alternariol; AME—alternariol monomethyl ether; 17-DMAG—17-dimethylaminoethylamino-17-demethoxygeldanamycin; 15-DON—15-acetyldeoxynivalenol; MEL—meleagrin; Neo—neosolaniol; 15ACS—15-acetoxyscirpenol; ENN A—enniatin A; ENN A1—enniatin A1; FB1—fumonisin B1; FB2—fumonisin B2; DON—deoxynivalenol; STC—sterigmatocystin; OTB—ochratoxin B; FUSX—fusarenon X; T-2—T-2 toxin; HT-2—HT-2 toxin; OTA—ochratoxin A; D3G—deoxynivalenol-3-glucoside; AFB1—aflatoxin B1; nd—not detected. Data are presented as means (n = 5) ± standard deviation (SD). a–i—means within a lines with different letters are significantly different ($p \leq 0.05$).
