# Supplementary material for: The Contribution of Extruded and Fermented Wheat Bran to the Quality Parameters of Wheat Bread, Including the Profile of Volatile Compounds and Their Relationship with Emotions Induced for Consumers
Source: Foods. 2021 Oct 18;10(10):2501. doi: 10.3390/foods10102501 (PMC8535695; doi:10.3390/foods10102501)
Supplement: Supplementary file 1 [file foods-10-02501-s001.zip › Supplementary file 2. Procedure of the FaceReader analysis.pdf]

Supplementary file 2. Procedure of the FaceReader analysis

The breads were tested (by the same 20 judges) by applying FaceReader 8.0 software (Noldus Information Technology, Wageningen, The Netherlands), with a scoring scale of eight emotion patterns (neutral, happy, sad, angry, surprised, scared, disgusted, contempt).

The whole procedure of the bread induced emotions of the judges was filmed with a Microsoft LifeCam Studio webcam, mounted on the laptop facing the participants, using Media Recorder (Noldus Information Technology, Wageningen, The Netherlands) software.

The recordings with a resolution of 640×480 at 25 frames per second were saved as AVI files and analyzed frame by frame with FaceReader 5 software, scaling the 8 basic emotion patterns (''neutral'', ''happy'', ''sad'', ''angry'', ''suprised'', ''scared'', ''disgusted'', ''contempt'').

For each sample, the section of intentional facial expression (exactly from the point when the subject had finished raising their hand to give the signal until the subject started lowering the hand again) was extracted and used for the statistical analysis. *FaceReader* contains an image quality bar, which gives a good indication of how well the program is able to model the face depicted in the image.

For the best image quality, the main attention was focused on camera position and illumination.

For this reason, participants were asked to sit and look frontally into the camera.

For the statistical analysis, the maximum values of the facial expression patterns of the respective section were used.
